# Supplementary material for: Resolving synaptic events using subsynaptically targeted GCaMP8 variants
Source: eLife. 2026 Mar 2;14:RP107939. doi: 10.7554/eLife.107939 (PMC12952790; doi:10.7554/eLife.107939)
Supplement: Supplementary file 1. — This table reports the full statistical details and properties for data presented in the indicated figures, including p-values, mean ± SEM, sample sizes (n), and genotypes for all conditions tested. Ca²⁺ imaging parameters include ΔF/F (or ΔR/R), rise time (τrise), and decay time (τdecay) constants. Electrophysiological parameters include mEPSP amplitude, EPSP amplitude, quantal content (QC), input resistance, and resting potential. p-values from one-way ANOVA with Tukey’s multiple comparison test are shown for key contrasts between genotypes and indicators. Data for outlier analysis, correlation analysis and linear regression analyses are included where applicable. [file elife-107939-supp1.docx]

**Supplementary File 1: Absolute values and statistical comparisons for Ca²⁺ imaging and electrophysiology data.**

This table reports the full statistical details and properties for data presented in the indicated figures, including p-values, mean ± SEM, sample sizes (n), and genotypes for all conditions tested. Ca²⁺ imaging parameters include ΔF/F (or ΔR/R), rise time (τ_rise_), and decay time (τ_decay_) constants. Electrophysiological parameters include mEPSP amplitude, EPSP amplitude, quantal content (QC), input resistance, and resting potential. p-values from one-way ANOVA with Tukey’s multiple comparison test are shown for key contrasts between genotypes and indicators. Data for outlier analysis, correlation analysis and linear regression analyses are included where applicable.

| **Figure** | **Label** | **Genotype** | **Motor Neuron** | **mEPSP amplitude (mV)** | **EPSP amplitude (mV)** | **QC** | **mEPSP frequency (Hz)** | **R input (MΩ)** | **Resting potential (mV)** | **n** | **P value (significance: mEPSP, EPSP, QC)** |
| --- | --- | --- | --- | --- | --- | --- | --- | --- | --- | --- | --- |
| Figure 1  -  figure supplement 1 | WT | *w^1118^* | Is + Ib | 0.995  (±0.048) | 30.96  (±1.182) | 30.34  (±2.101) | 3.133  (±0.319) | 12.11 (±0.138) | 65.38  (±1.19) | 10 | - |
|  | OK319> Syt::GCaMP6s | *w;OK319-GAL4/+; UAS-Syt::GCaMP6s/+* | Is + Ib | 0.996  (±0.057) | 29.39  (±1.035) | 29.88  (±1.024) | 4.067  (±0.509) | 12.89 (±0.197) | 65.46  (±1.541) | 8 | >0.9999 (ns),  0.8985 (ns),  0.9997(ns),  0.9694 (ns) |
|  | OK319> Scar8f | *w;OK319-GAL4/+; UAS-Syt::mScarlet::GCaMP8f/+* | Is + Ib | 1.057  (±0.053) | 22.33  (±0.941) | 21.02  (±1.488) | 2.222  (±0.4923) | 12.71 (±0.126) | 66.62  (±1.284) | 9 | 0.7858 (ns),  <0.0001 (****),  <0.0001 (***),  0.8299 (ns) |
|  | OK319> Scar8m | *w;OK319-GAL4/+; UAS-Syt::mScarlet3::GCaMP8m/+* | Is + Ib | 1.031  (±0.027) | 28.61  (±2.122) | 27.89  (±2.289) | 2.692  (±0.187) | 10.87 (±0.108) | 66.23  (±1.105) | 8 | 0.9867 (ns),  0.5656 (ns),  0.7704 (ns),  0.9940 (ns) |
|  | OK6> Bar8f | *w;OK6-GAL4/+; BRP::mScarlet::GCaMP8f/+* | Is + Ib | 1.088  (±0.018) | 32.30  (±0.425) | 29.73  (±0.595) | 2.117  (±0.611) | 11.25 (±0.143) | 63.62  (±1.443) | 8 | 0.3526 (ns),  0.9535 (ns),  >0.9999 (ns),  0.6383 (ns) |
|  | OK6> Bar8m | *w;OK6-GAL4/+; BRP::mScarlet3::GCaMP8m/+* | Is + Ib | 0.9278  (±0.051) | 31.97  (±0.731) | 35.73  (±2.422) | 2.824  (±0.428) | 13.51 (±0.163) | 66.23  (±1.835) | 8 | 0.7966 (ns),  0.9932 (ns),  0.1136 (ns),  0.9870 (ns) |
|  | SynapGCaMP6f | *w;MHC-CD8-GCaMP6f-Sh;+* | Is + Ib | 1.043  (±0.025) | 29.69  (±0.934) | 28.60  (±1.011) | 3.606  (±0.608) | 11.84 (±0.198) | 65.13  (±1.484) | 8 | 0.9317 (ns),  0.9646 (ns),  0.9495 (ns),  >0.9999 (ns) |
|  | SynapGCaMP8f | *w;;MHC-CD8-GCaMP8f-Sh* | Is + Ib | 1.124  (±0.013) | 29.55  (±0.736) | 26.34  (±0.781) | 2.692  (±0.390) | 13.97 (±0.160) | 66.51  (±1.471) | 8 | 0.0825 (ns),  0.9395 (ns),  0.2607 (ns),  0.9839 (ns) |
|  | SynapGCaMP8m | *w**;;MHC-CD8-GCaMP8m-Sh* | Is + Ib | 1.007  (±0.024) | 29.73  (±0.901) | 28.60  (±1.011) | 3.587  (±0.546) | 11.90 (±0.105) | 67.39  (±1.276) | 8 | >0.9999 (ns),  0.9702 (ns),  0.9998 (ns),  >0.9999 (ns) |

| **Figure** | **Label** | **Genotype** | **Motor Neuron** | **△F/F**  **(△R/R)** | **p value** | **Τau rise (msec)** | **p value** | **Τau decay (msec)** | **p value** | **n** |
| --- | --- | --- | --- | --- | --- | --- | --- | --- | --- | --- |
| 3B | OGB-1 | *w^1118^* | Ib | 0.227  (±0.020) | 0.0279  (*) | 3.040  (±0.291) | 0.7398  (ns) | 60.13  (±3.259) | <0.0001  (****) | 7 |
| 3B | Scar8f | *w;OK6-GAL4/+; UAS-Syt::mScarlet::GCaMP8f/+* | Ib | 0.322  (±0.032) |  | 2.893  (±0.323) |  | 38.52  (±1.053) |  | 7 |
| 3D | RSET-GCaMP8m | *w;OK319-GAL4/+; UAS-RSET-GCaMP8m/+* | Ib | 0.397  (±0.029) | <0.0001  (****) | 6.933  (±0.454) | >0.9999  (ns) | 137.8  (±6.887) | 0.2679  (ns) | 7 |
| 3D | Scar8m | *w;OK319-GAL4/+; UAS-Syt::mScarlet3::GCaMP8m/+* | Ib | 0.6765  (±0.018) |  | 6.736  (±0.452) |  | 100.8  (±5.135) |  | 13 |

| **Figure** | **Label** | **Genotype** | **Motor Neuron** | **△F/F**  **(△R/R)** | **p value** | **Frequency faciliation** | **p value** | **Τau decay (msec)** | **p value** | **n** |
| --- | --- | --- | --- | --- | --- | --- | --- | --- | --- | --- |
| 3E | OGB-1 (train-evoked) |  | Ib | 0.441  (±0.031) | 0.0279  (*) | 1.976  (±0.118) | <0.0001  (****) | 107.8  (±8.875) | 0.1164  (ns) | 7 |
| 3E | Scar8f (train-evoked) | *w;OK6-GAL4/+; UAS-Syt::mScarlet::GCaMP8f/+* | Ib | 0.919  (±0.085) |  | 3.121  (±0.1169) |  | 132.2  (±11.34) |  | 7 |

| **Figure** | **Label** | **Genotype** | **Motor Neuron** | **△F/F**  **(△R/R)** | **p value**  **(6s vs 8f, 6s vs 8m, 8f vs 8m)** | **Τau rise (msec)** | **p value**  **(6s vs 8f, 6s vs 8m, 8f vs 8m)** | **Τau decay (msec)** | **p value**  **(6s vs 8f, 6s vs 8m, 8f vs 8m)** | **n** |
| --- | --- | --- | --- | --- | --- | --- | --- | --- | --- | --- |
| 4E | Syt::GCaMP6s | *w;OK319-GAL4/+; UAS-Syt::GCaMP6s/+* | Ib | 0.138  (±0.004) | <0.0001  (****), <0.0001  (****), <0.0001  (****) | 43.77  (±3.134) | <0.0001  (****), <0.0001  (****), 0.7827  (ns) | 171.7  (±8.01) | <0.0001  (****), <0.0001  (****), 0.0046  (**) | 14 |
| 4E | Scar8f | *w;OK319-GAL4/+; UAS-Syt::mScarlet::GCaMP8f/+* | Ib | 0.389  (±0.010) |  | 5.265  (±1.165) |  | 66.64  (±1.849) |  | 15 |
| 4E | Scar8m | *w;OK319-GAL4/+; UAS-Syt:: mScarlet3::GCaMP8m/+* | Ib | 0.632  (±0.011) |  | 6.984  (±0.604) |  | 99.20  (±3.785) |  | 14 |

| **Figure** | **Label** | **Genotype** | **Motor Neuron** | **△F/F**  **(△R/R)** | **p value** | **n** |
| --- | --- | --- | --- | --- | --- | --- |
| 5D | Scar8m/WT Ib | *w;OK319-GAL4/+; UAS-Syt:: mScarlet3::GCaMP8m/+* | Ib | 0.590  (±0.048) | <0.0001  (****) | 9 |
| 5D | Scar8m/WT Is | *w;OK319-GAL4/+; UAS-Syt:: mScarlet3::GCaMP8m/+* | Is | 1.100  (±0.060) |  | 9 |
| 5F | Scar8m/WT Ib | w;OK319-GAL4/+; UAS-Syt:: mScarlet3::GCaMP8m/+ | Ib | 0.605  (±0.042) | <0.0001  (****) | 9 |
| 5F | GluRIIA^-/-^ Ib | *W;OK371,GluRIIA^pv3^/GluRIIA^pv3^; UAS-Syt:: mScarlet3::GCaMP8m/+* | Ib | 0.941  (±0.035) |  | 12 |

| **Figure** | **Label** | **Genotype** | **Motor Neuron** | **△F/F**  **(△R/R)** | **p value**  **(8f vs 8m area,**  **8f vs 8m line,**  **8m line vs 8m area)** | **Τau rise (msec)** | **p value**  **(8f vs 8m area,**  **8f vs 8m line,**  **8m line vs 8m area)** | **Τau decay (msec)** | **p value**  **(8f vs 8m area,**  **8f vs 8m line,**  **8m line vs 8m area)** | **n** |
| --- | --- | --- | --- | --- | --- | --- | --- | --- | --- | --- |
| 6D | Bar8f | *w;OK6-GAL4/+; BRP::mScarlet::GCaMP8f/+* | Ib | 1.904  (±0.298) | <0.0001  (****), 0.357,  (ns),  <0.0001  (****) | 9.396  (±2.151) | 0.822  (ns),  <0.0001  (****), <0.0001  (****) | 22.71  (±18.553) | <0.0001  (****), <0.0001  (****), 0.134  (ns) | 8 |
| 6D | Bar8m (area scan) | *w;OK6-GAL4/+; BRP::mScarlet::GCaMP8m/+* | Ib | 3.280  (±0.415) |  | 9.019 (±2.575) |  | 47.97 (±10.973) |  | 7 |
| 6D | Bar8m (line scan) | *w;OK6-GAL4/+; BRP::mScarlet::GCaMP8m/+* | Ib | 2.051  (±0.503) |  | 3.230 (±1.471) |  | 40.26  (±9.812) |  | 8 |

**Figure 6E (left):** Data represent mean ± SEM of ΔR/R values for Resonant area scan of 30 active zones from Bar8f-expressing NMJs (genotype: *w;OK6-GAL4/+; BRP::mScarlet::GCaMP8f/+*) (n=8 NMJ). Outlier status is determined using the interquartile range (IQR) method on individual data points: Q1 = 1.454 (25th percentile), Q3 = 2.268 (75th percentile). Mean intensities of columns 27–30 exceed the upper bound and are marked as outliers.

| **AZ#** | **1** | **2** | **3** | **4** | **5** | **6** | **7** | **8** | **9** | **10** |
| --- | --- | --- | --- | --- | --- | --- | --- | --- | --- | --- |
| **△R/R** | 1.504 | 1.513 | 1.549 | 1.566 | 1.644 | 1.661 | 1.691 | 1.697 | 1.701 | 1.720 |
| **Std. Deviation** | 0.07174 | 0.1498 | 0.1143 | 0.1017 | 0.1038 | 0.08544 | 0.1460 | 0.08549 | 0.09921 | 0.1067 |
| **Outlier** | No | No | No | No | No | No | No | No | No | No |
| **AZ#** | 10 | 12 | 13 | 14 | 15 | 16 | 17 | 18 | 19 | 20 |
| **△R/R** | 1.755 | 1.759 | 1.785 | 1.787 | 1.807 | 1.824 | 1.896 | 1.900 | 1.907 | 1.925 |
| **Std. Deviation** | 0.1229 | 0.1049 | 0.1203 | 0.1668 | 0.1528 | 0.06702 | 0.1379 | 0.1195 | 0.1791 | 0.1929 |
| **Outlier** | No | No | No | No | No | No | No | No | No | No |
| **AZ#** | 21 | 22 | 23 | 24 | 25 | 26 | 27 | 28 | 29 | 30 |
| **△R/R** | 2.023 | 2.062 | 2.072 | 2.127 | 2.134 | 2.184 | 2.377 | 2.413 | 2.425 | 2.706 |
| **Std. Deviation** | 0.2157 | 0.1229 | 0.1357 | 0.1389 | 0.1387 | 0.1848 | 0.2230 | 0.1628 | 0.1804 | 0.1372 |
| **Outlier** | No | No | No | No | No | No | Yes | Yes | Yes | Yes |

**Figure 6E (middle):** Data represent mean ± SEM of ΔR/R values for Resonant area scan of 30 active zones from Bar8m-expressing NMJs (genotype: *w;OK6-GAL4/+; BRP::mScarlet::GCaMP8m/+*) (n=7 NMJ). Outlier status is determined using the interquartile range (IQR) method on individual data points: Q1 = 2.71 (25th percentile), Q3 = 3.83 (75th percentile). The mean intensity values in columns 1, 28, 29, and 30 exceed the upper bound and are marked as outliers.

| **AZ#** | **1** | **2** | **3** | **4** | **5** | **6** | **7** | **8** | **9** | **10** |
| --- | --- | --- | --- | --- | --- | --- | --- | --- | --- | --- |
| **△R/R** | 2.570 | 2.711 | 2.718 | 2.777 | 2.908 | 2.920 | 2.962 | 3.006 | 3.008 | 3.011 |
| **Std. Deviation** | 0.1135 | 0.1574 | 0.09472 | 0.4138 | 0.08930 | 0.1761 | 0.1111 | 0.09609 | 0.1770 | 0.3050 |
| **Outlier** | Yes | No | No | No | No | No | No | No | No | No |
| **AZ#** | 10 | 12 | 13 | 14 | 15 | 16 | 17 | 18 | 19 | 20 |
| **△R/R** | 3.014 | 3.023 | 3.055 | 3.222 | 3.275 | 3.312 | 3.338 | 3.344 | 3.372 | 3.423 |
| **Std. Deviation** | 0.2792 | 0.2036 | 0.2087 | 0.2315 | 0.1654 | 0.2702 | 0.1639 | 0.1447 | 0.1348 | 0.2328 |
| **Outlier** | No | No | No | No | No | No | No | No | No | No |
| **AZ#** | 21 | 22 | 23 | 24 | 25 | 26 | 27 | 28 | 29 | 30 |
| **△R/R** | 3.438 | 3.481 | 3.529 | 3.587 | 3.624 | 3.692 | 3.831 | 3.935 | 4.146 | 4.160 |
| **Std. Deviation** | 0.2824 | 0.2898 | 0.2229 | 0.2349 | 0.2161 | 0.2328 | 0.1887 | 0.2629 | 0.2488 | 0.2867 |
| **Outlier** | No | No | No | No | No | No | No | Yes | Yes | Yes |

**Figure 6E (right):** Data represent mean ± SEM of ΔR/R values for Galvano line scan of 30 active zones from Bar8m-expressing NMJs (genotype: *w;OK6-GAL4/+; BRP::mScarlet::GCaMP8m/+*) (n=8 NMJ). Outlier status is determined using the interquartile range (IQR) method on individual data points: Q1 = 1.65 (25th percentile), Q3 = 2.46 (75th percentile). The mean intensity values in column 1, 28, 29, 30 exceeds the upper bound and is marked as outliers.

| **AZ#** | **1** | **2** | **3** | **4** | **5** | **6** | **7** | **8** | **9** | **10** |
| --- | --- | --- | --- | --- | --- | --- | --- | --- | --- | --- |
| **△R/R** | 1.137 | 1.377 | 1.584 | 1.627 | 1.642 | 1.642 | 1.657 | 1.677 | 1.709 | 1.752 |
| **Std. Deviation** | 0.02965 | 0.02603 | 0.04381 | 0.05424 | 0.02733 | 0.07045 | 0.04215 | 0.04927 | 0.04375 | 0.04886 |
| **Outlier** | Yes | Yes | Yes | Yes | Yes | Yes | No | No | No | No |
| **AZ#** | 10 | 12 | 13 | 14 | 15 | 16 | 17 | 18 | 19 | 20 |
| **△R/R** | 1.782 | 1.803 | 1.803 | 1.814 | 1.865 | 1.917 | 1.958 | 2.107 | 2.114 | 2.127 |
| **Std. Deviation** | 0.04090 | 0.04185 | 0.04185 | 0.03195 | 0.04062 | 0.05031 | 0.06363 | 0.1014 | 0.09142 | 0.07513 |
| **Outlier** | No | No | No | No | No | No | No | No | No | No |
| **AZ#** | 21 | 22 | 23 | 24 | 25 | 26 | 27 | 28 | 29 | 30 |
| **△R/R** | 2.210 | 2.265 | 2.320 | 2.455 | 2.539 | 2.712 | 2.914 | 2.939 | 3.027 | 3.064 |
| **Std. Deviation** | 0.09016 | 0.07923 | 0.08218 | 0.04884 | 0.08143 | 0.07516 | 0.1091 | 0.09999 | 0.07484 | 0.1087 |
| **Outlier** | No | No | No | No | Yes | Yes | Yes | Yes | Yes | Yes |

| **Figure** | **Label** | **Genotype** | **Motor Neuron** | **Metric (Y vs X)** | **Equation** | **Pearson's r** | **R²** | **p-value (correlation)** | **n** |
| --- | --- | --- | --- | --- | --- | --- | --- | --- | --- |
| Figure 6-figure supplement 1 | Bar8f | *w;OK6-GAL4/+; BRP::mScarlet::GCaMP8f/+* | Ib | ΔR/R vs. AZ Size | Y = -0.8644*X + 2.355 | -0.4902 | 0.2403 | 0.0069 (**) | 29 |
|  | Bar8f | *w;OK6-GAL4/+; BRP::mScarlet::GCaMP8f/+* | Ib | Sum ΔF vs. AZ Size | Y = 259.8*X + 40.40 | 0.7316 | 0.5353 | <0.0001 (****) | 29 |

| **Figure** | **Label** | **Genotype** | **Motor Neuron** | **△F/F** | **p value**  **(6s vs 8f,**  **6s vs 8m,**  **8f vs 8m)** | **Τau rise (msec)** | **p value**  **(6s vs 8f,**  **6s vs 8m,**  **8f vs 8m)** | **Τau decay (msec)** | **p value**  **(6s vs 8f,**  **6s vs 8m,**  **8f vs 8m)** | **n** |
| --- | --- | --- | --- | --- | --- | --- | --- | --- | --- | --- |
| 7E | SynapGCaMP6f | *w;MHC-CD8-GCaMP6f-Sh;+* | Ib | 0.271  (±0.024) | 0.2164 (ns), <0.0001  (****), <0.0001  (****) | 21.09  (±2.514) | 0.0182 (*), 0.0070 (**), 0.9944 (ns) | 98.76  (±6.914) | <0.0001  (****), <0.0001  (****), 0.0036  (**) | 47 |
| 7E | SynapGCaMP8f | *w;;MHC-CD8-GCaMP8f-Sh* | Ib | 0.349  (±0.030) |  | 14.04  (±1.190) |  | 41.98  (±1.829) |  | 50 |
| 7E | SynapGCaMP8m | *w;;MHC-CD8-GCaMP8m-Sh* | Ib | 0.575  (±0.043) |  | 14.22  (±0.985) |  | 67.35  (±1.640) |  | 58 |

| **Figure** | **Label** | **Genotype** | **Motor Neuron** | **% mEPSPs detected as Ca²⁺ minis** | **p value**  **(6s vs 8f, 6s vs 8m, 8f vs 8m)** | **n** |
| --- | --- | --- | --- | --- | --- | --- |
| 8B | SynapGCaMP6f | *w;MHC-CD8-GCaMP6f-Sh;+* | Ib | 56.87  (±7.217) | <0.0001 (****), <0.0001 (****),  0.5645 (ns) | 12 |
| 8B | SynapGCaMP8f | *w;;MHC-CD8-GCaMP8f-Sh* | Ib | 87.55  (±1.538) |  | 17 |
| 8B | SynapGCaMP8m | *w;;MHC-CD8-GCaMP8m-Sh* | Ib | 93.08  (±1.318) |  | 12 |

| **Figure** | **Label** | **Genotype** | **Motor Neuron** | **Metric (Y vs X)** | **Equation** | **Pearson's r** | **R²** | **p-value (correlation)** | **n** |
| --- | --- | --- | --- | --- | --- | --- | --- | --- | --- |
| 8C | SynapGCaMP6f | *w;MHC-CD8-GCaMP6f-Sh;+* | Ib | Ca^2+^ mini ΔF/F vs. mEPSP (mV) | Y = 0.1231*X + 0.08230 | 0.4579 | 0.2097 | <0.0001 (****) | 83 |
| 8C | SynapGCaMP8f | *w;;MHC-CD8-GCaMP8f-Sh* | Ib | Ca^2+^ mini ΔF/F vs. mEPSP (mV) | Y = 0.2910*X + 0.1663 | 0.7316 | 0.5374 | <0.0001 (****) | 98 |
| 8D | SynapGCaMP8m | *w;;MHC-CD8-GCaMP8m-Sh* | Ib | Ca^2+^ mini ΔF/F vs. mEPSP (mV) | Y = 0.4216*X + 0.1672 | 0.8098 | 0.6557 | <0.0001 (****) | 97 |

| **Figure** | **Label** | **Genotype** | **Motor Neuron** | **mEPSP amplitude (mV)** | **mEPSP frequency (Hz)** | **R input (MΩ)** | **Resting potential (mV)** | **P Value (significance: mEPSP, mEPSP freq)** | **n** |
| --- | --- | --- | --- | --- | --- | --- | --- | --- | --- |
| 8E | WT | *w;;Is-GAL4/UAS-BoNT-C* | Ib | 0.636  (±0.015) | 1.062  (±0.100) | 11.91  (±0.115) | 64.45  (±1.561) | - | 41 |
| 8E | IIB^-/-^ | *w;GluRIIB^sp5^;Is-GAL4/UAS-BoNT-C* | Ib | 0.824  (±0.017) | 2.433  (±0.147) | 12.89 (±0.197) | 65.46  (±1.541) | <0.0001 (****),  <0.0001 (****) | 41 |
| 8E | IIA^-/-^ | *w;GluRIIA^pv3^;Is-GAL4/UAS-BoNT-C* | Ib | 0.383  (±0.007) | 0.715  (±0.111) | 12.11 (±0.138) | 65.38  (±1.19) | <0.0001 (****),  <0.0001 (****) | 41 |

| **Figure** | **Label** | **Genotype** | **Motor Neuron** | **△F/F** | **p value**  **(WT vs IIB-/-, WT vs IIA-/-, IIB-/- vs IIA-/-)** | **n** |
| --- | --- | --- | --- | --- | --- | --- |
| 8E | WT | *w;;MHC-CD8-GCaMP8m-Sh* | Ib | 0.713  (±0.021) | <0.0001 (****), <0.0001 (****),  <0.0001 (****) | 65 |
| 8E | IIB^-/-^ | *W;GluRIIBsp5;MHC-CD8-GCaMP8m-Sh* | Ib | 0.967  (±0.036) |  | 69 |
| 8E | IIA^-/-^ | *W;GluRIIBsp5;MHC-CD8-GCaMP8m-Sh* | Ib | 0.427  (±0.017) |  | 59 |

**Kolmogorov–Smirnov Test Results for Mini Amplitude Distributions for MN-Ib**

D statistic: Maximum difference between cumulative distributions.

All comparisons reached p < 0.001, indicating significantly different mini amplitude distributions.

| **Figure** | **Label** | **Genotype** | **Motor Neuron** | **mEPSP amplitude (mV)** | **D statistic (WT vs IIB-/-, WT vs IIA-/-, IIB-/- vs IIA-/-)** | **P Value (WT vs IIB-/-, WT vs IIA-/-, IIB-/- vs IIA-/-)** | **n** |
| --- | --- | --- | --- | --- | --- | --- | --- |
| 8F | WT | *w;;Is-GAL4/UAS-BoNT-C* | Ib | 0.636  (±0.015) | 0.1854,  0.3647,  0.4043 | <0.0001 (****), <0.0001 (****),  <0.0001 (****) | 41 |
| 8F | IIB^-/-^ | *w;GluRIIB^sp5^;Is-GAL4/UAS-BoNT-C* | Ib | 0.824  (±0.017) |  |  | 41 |
| 8F | IIA^-/-^ | *w;GluRIIA^pv3^;Is-GAL4/UAS-BoNT-C* | Ib | 0.383  (±0.007) |  |  | 41 |

| **Figure** | **Label** | **Genotype** | **Motor Neuron** | **△F/F** | **D statistic (WT vs IIB-/-, WT vs IIA-/-, IIB-/- vs IIA-/-)** | **p value**  **(WT vs IIB-/-, WT vs IIA-/-, IIB-/- vs IIA-/-)** | **n** |
| --- | --- | --- | --- | --- | --- | --- | --- |
| 8F | WT | *w;;MHC-CD8-GCaMP8m-Sh* | Ib | 0.713  (±0.021) | 0.2046,  0.2938,  0.3277 | <0.0001 (****), <0.0001 (****),  <0.0001 (****) | 65 |
| 8F | IIB^-/-^ | *W;GluRIIB^sp5^;MHC-CD8-GCaMP8m-Sh* | Ib | 0.967  (±0.036) |  |  | 69 |
| 8F | IIA^-/-^ | *W;GluRIIB^sp5^;MHC-CD8-GCaMP8m-Sh* | Ib | 0.427  (±0.017) |  |  | 59 |
